# Prenatal and post-natal cost of small for gestational age infants: a national study

**DOI:** 10.1186/s12913-017-2155-x

**Published:** 2017-03-21

**Authors:** Alicia Marzouk, Antoine Filipovic-Pierucci, Olivier Baud, Vassilis Tsatsaris, Anne Ego, Marie-Aline Charles, François Goffinet, Danièle Evain-Brion, Isabelle Durand-Zaleski

**Affiliations:** 1 URC ECO, Hôtel Dieu 1 Place du Parvis de Notre Dame, 75004 Paris, France; 2 PREMUP Foundation, Paris, France; 3 Robert Debré hospital, Paris, France, Inserm U1141, Paris Diderot University, Paris, France; 4 Port-Royal maternity, DHU Risk in Pregnancy, Cochin Broca Hôtel-Dieu hospital, INSERM Unité 1139, Paris-Descartes University, Paris, France; 5 Grenoble Alpes University, TIMC-IMAG, CNRS, Grenoble, France; 6 Obstetrical, Perinatal and Pediatric Epidemiology Research Team (EPOPé), Research Center for Epidemiology and Biostatistics Sorbonne Paris Cité (CRESS), Paris Descartes University, Paris, France; 7 AP HP Henri Mondor hospital, Créteil, France; 8 INSERM UMRS 1123 (ECEVE), Paris, France; 9 Paris Est university, Créteil, France; 10 INSERM U1153, Early origin of the child’s health and development team (ORCHAD), Research Center for Epidemiology and Biostatistics Sorbonne Paris Cité (CRESS), Paris Descartes University, Paris, France

**Keywords:** Small for gestational age, Database, Economic cost, Prematurity

## Abstract

**Background:**

Small for gestational age (SGA) infants are at increased risk for preterm birth morbidities as well as a range of adverse perinatal outcomes that result in part from associated premature birth. We sought to evaluate the costs of SGA versus appropriate for gestational age (AGA) infants in France from pregnancy through the first year of life and separate the contributions of prematurity from the contribution of foetal growth on costs.

**Methods:**

This is a cross-sectional population-based study using national hospital discharge data from French public and private hospitals. SGA infants were defined as newborns with a birth weight below the 10th percentile of French intrauterine growth curves adjusted for foetal sex. AGA infants were defined as newborns with a birth weight between the 25th and the 75th. All births were selected between January 1st, 2011 and December 31st, 2011. Costs were calculated from the hospital perspective for both mothers and children using their diagnostic related group and the French national cost study. Hospital outcomes were extracted from the database and compared by gestational age and mode of delivery.

**Results:**

Of 777,720 total births in 2011, 84,688 SGA births (10.9%) and 395,760 AGA births (50.8%) were identified. After adjustment for gestational age, the cost for an SGA infant was €2,783 higher than for an AGA infant. The total maternal and infant hospital cost of SGA in France was estimated at 23% the total cost for deliveries. The high cost is explained by higher complication rates, more frequent hospital readmissions and longer lengths of stay.

**Conclusions:**

Being small for gestational age is an independent contributor to 1-year hospital costs for both mothers and infants.

## Background

Small for gestational age (SGA) infants are defined by a birth weight lower than the 10th percentile for a given gestational age. SGA infants are at increased risk for preterm birth morbidities as well as a range of adverse perinatal outcomes that result in part from associated premature birth. In adulthood, conditions such as metabolic syndrome, diabetes and hypertension develop more often in individuals born SGA regardless of whether the birth was preterm. Although the health sequelae of SGA are well-documented, relatively little is known about their economic consequences either in early or later life [1]. Morbidity and mortality related to SGA creates a burden for both families and the health care system.

Several national and international studies have addressed the cost of preterm birth; however, few have estimated the cost of SGA separately from the cost of prematurity [2–6]. A Canadian study suggested that gestational age was a better predictor of cost than fetal growth: premature SGA babies cost at birth was $109,286 versus $85,103 for premature non-SGA babies, while the average cost of full term SGA baby was about twice the cost of a full term non-SGA baby [2]. The intricacy of the relationship between prematurity and SGA contributes to the difficulty of distinguishing those two pathologies in the current literature [3, 4, 7]. Such a distinction, however, would allow better assessment of the medical and economic consequences of SGA during the neonatal period, childhood and throughout life and help to focus research priorities on early identification of at-risk foetuses.

Our objective was to estimate the additional hospital cost associated with SGA infants from pregnancy through the first year of life and to separate the contributions of prematurity from the contribution of SGA on costs.

## Methods

We used data from the 2011 and 2012 French national hospital claims database (*Programme de Médicalisation des Systèmes d’Information*; PMSI), which collects linked, anonymised medical records of all French inpatient and day case admissions in both public and private hospitals. Maternal and newborn records are linked at the national level. Such administrative databases have been previously used to estimate costs and outcomes of preterm infants [4, 8, 9].

### Population

We extracted the records of all infants born in metropolitan France in public and private hospitals from January 1, 2011 to December 31, 2011. Hospitalisations during the first year of life were identified by record linkage. Selection of the population was based on the Diagnostic Related Group (DRG), weight and gestational age in addition to diagnoses, procedures and administrative information.

Exclusion criteria were multiple pregnancies, stillbirths (because postnatal costs would be non-existent), problems in the anonymisation process, unclassifiable DRGs, congenital or chromosomal abnormality and absence of data about the weight, sex and gestational age.

We defined subgroups by gestational age (in completed weeks): [22–28[ (from 22 to 28 not included), [28–32[, [32–37[, [37–39[ and [39–43[. Births before 37 weeks were considered preterm. Within each class of gestational age we classified newborns into SGA and AGA. SGA infants were defined as having birthweights below the 10th percentile of the French intrauterine growth curve adjusted for foetal sex. [10] AGA infants were defined as having birthweights between the 25th and the 75th percentiles of the same growth curve. We chose this definition for the control group in order to reduce the variability in practice patterns and costs that would occur in the range approaching pathological conditions. These reference curves were recently developed using the representative sample of births from the French Perinatal Survey (FPS) 2010. Initially proposed by Gardosi in the 1990s, they are based on a modelisation of intrauterine growth and foetal weight. SGA thresholds are individually defined according to foetal and maternal physiological factors influencing growth. This definition of SGA births is currently supported by several international guidelines for screening and management of SGA births. Due to a lack of data about maternal height, weight and parity in the French hospital discharge database, we applied the model adjusted for foetal sex. [10–12] Mothers were identified in the database by linkage to the newborn hospital records. Data analysis was authorized by the French data protection authority (CNIL authorization number: 1606292 v 1).

### Outcomes

Type of delivery and complications were identified by the International Classification of Diseases, tenth revision (ICD-10) and procedures codes. Mortality was defined as death during the index hospitalisation or during the first year of life and excluded stillbirths. Deaths were identified using the discharge status; in-hospital death during the first year was identified by record linkage. The mortality information provided by DRGs was verified via a national standard procedure with linkage to national death registries [13–15].

### Hospital resources and costs

A summary of all hospital inpatient service utilisation from the day of birth through the first year of life was compiled for all study infants. Record linkage between newborns and mothers was used to identify hospital admissions during pregnancy and delivery-related costs.

Data extracted included mode of delivery and type of neonatology unit, date of each admission, length of stay, procedures performed and ICD-10 codes. The total time infants and mothers spent in the hospital was calculated by summing the lengths of stay of successive admissions (including day cases) over a 1-year period. Hospital costs were calculated from the health insurance perspective [16]. Ambulatory expenses are not included in the discharge database. The total cost of deliveries in France and the SGA share of this cost was also estimated. All costs are in 2015 €.

### Statistical analysis

Maternal and neonatal characteristics, resource utilisation (length of stay, use of intensive care, readmission) and costs were summarised by gestational age and compared between SGA and AGA infants using Pearson’s Chi-squared test and Wilcoxon’s test for qualitative and quantitative variables respectively. All tests were two-sided, and *p*-values lower than 0.05 were considered statistically significant. Determinants of cost variations between the two groups were studied according to gestational age. Results are presented in means and standard deviations (SD); because of the skewed distribution of costs we also reported medians and interquartile ranges (IQR). The excess mortality was calculated by dividing the mortality rate of the SGA group by the mortality rate of the AGA group in each gestational age class. Cost differences and their standard deviations were calculated using 1,000 bootstrap replications. All analyses were performed with SAS version 9.3 (SAS Institute, Cary, NC).

## Results

### Population

**Fig. 1:**
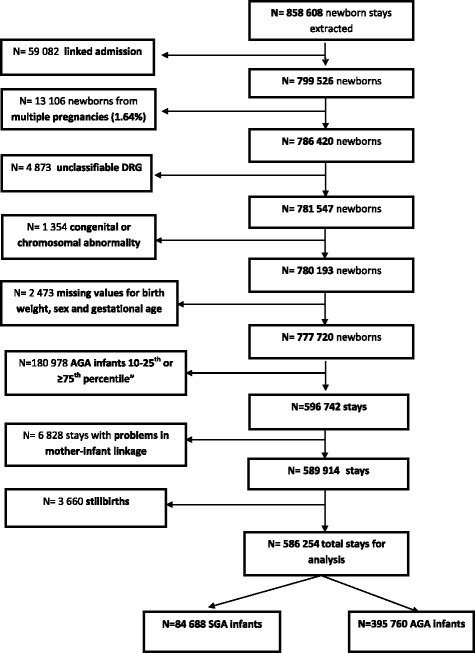
Selection of the study population

A total of 777,720 births and 858,608 admissions were identified nationally We excluded *N* = 59,082 linked admissions, *N* = 13,106 multiple pregnancies, *N* = 4,873 unclassifiable DRGs, *N* = 1,354 congenital or chromosomal abnormalities, *N* = 2,473 missing values for weight and gestational age, *N* = 180,978 non-SGA or -AGA births, *N* = 6,828 stays with problems in mother-infant record linkage and *N* = 3,660 stillbirths (Fig. 1). After applying the exclusion criteria, 84,688 SGA births (10.9% of total births) and 395,760 AGA births (51.8% of total births) remained for the analyses. There were 3,660 stillbirths, 2,069 in the SGA group (1.8% of total SGA births) versus 1,591 in the AGA group (0.3% of total AGA births).

Population characteristics are presented in Table 1. Preterm birth was reported for 11,097 individuals (13.1%) in the SGA group and 22,627 individuals (5.7%) in the AGA group (gestational age < 37 weeks). The average age of mothers was 28.9 (SD = 5.9) years in the SGA group and 29.1 (SD = 5.7) years in the AGA group (*p* < 0.001). The proportion of deliveries in public hospitals was higher in the SGA group (64,117 births, 75.7% versus 282,180 births, 71.3%).

**Table 1:** Characteristics of the population at birth in small and average for gestational age infants, and risk ratios between groups

	SGA *N* = 84,688		AGA *N* = 395,760		Risk Ratio AGA/SGA
	N	%	N	%	RR [CI 95%]
Sex					
Male	42,646	50.36	200,653	50.70	
Female	42,042	49.64	195,107	49.30	0.99 [0.97; 1.01]
Gestational age					
<37 weeks	11,097	13.10	22,627	5.72	0.53 [0.52; 0.53]
Delivery					
Caesarean	15,074	17.8	53,428	13.5	
Vaginal delivery	69,614	82.2	342,332	86.5	1.39 [1.35; 1.41]
ICU admission					
YES	18,320	21.63	28,485	7.20	0.31 [0.31; 0.32]

*ICU* Intensive Care Unit; *SGA* Small for Gestational Age; *AGA* Appropriate for Gestational Age

### Outcomes

Caesarean section delivery was performed for 17.8% of the SGA mothers versus 13.5% of the AGA mothers. The rate of caesarean section was consistently higher in SGA versus AGA births for all gestational ages, with the highest excess rate in the [28–32[ weeks gestational age group and lowest in the [37–39] weeks gestational age group (Table 2).

**Table 2:** Type of delivery section according to gestational age in SGA and AGA infants

		SGA delivery		AGA Delivery		Risk Ratio for caesarean section AGA/SGA
		Caesarean	Vaginal delivery	Caesarean	Vaginal delivery	RR [95% CI]
Gestational age (weeks)	[22–28[ (*N* = 1,213)	34.0%	66.0%	22.6%	77.4%	1.75 [1.37; 2.27]
	[28–32[ (*N* = 3,346)	46.7%	53.3%	29.7%	70.3%	2.08 [1.79; 2.44]
	[32–37[ (*N* = 29,165)	35.3%	64.7%	17.9%	82.1%	2.5 [2.38; 2.63]
	[37–39[ (*N* = 101,974)	19.9%	80.1%	15.9%	84.1%	1.32 [1.27; 1.37]
	[39–43[ (*N* = 344,750)	14.8%	85.2%	12.1%	87.9%	1.25 [1.22; 1.28]

*SGA* Small for Gestational Age; *AGA* Appropriate for Gestational Age

**Fig. 2:**
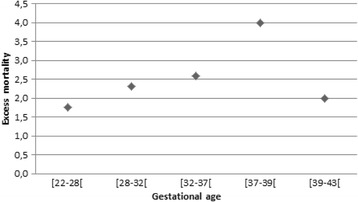
Excess mortality for SGA infants compared to AGA infants by to gestational age

Mortality during the first year of life was higher in SGA infants with 529 deaths (0.60%) versus 628 deaths (0.16%) in AGA, *p* < 0.001. Mortality was highest in the [22–28[ weeks gestational age group (Table 3) regardless of birth weight. After stratification by gestational age, we found that the excess mortality of SGA was the lowest between 22 and 28 weeks of gestation and increased twofold after 37 weeks (Fig. 2).

**Table 3:** SGA and AGA total 1-year mortality by gestational age (stillbirths excluded)

	SGA		TOTAL population	AGA		TOTAL population
Gestational age at delivery (weeks)	*N* deaths	%	*N*	*N* deaths	%	*N*
[22–28 [	187	36.2	517	223	32.0	696
[28–32 [	108	6.0	1,791	66	4.2	1,555
[32–37 [	96	1.1	8,789	99	0.5	20,376
[37–39 [	61	0.3	17,491	79	0.09	84,483
[39–43[	77	0.1	56,100	161	0.06	288,650
TOTAL	529	0.6	84,688	628	0.16	395,760

*SGA* Small for Gestational Age; *AGA* Appropriate for Gestational Age

### Hospital resources and costs

#### Mothers

Hospital admission during the prenatal period occurred in 23.6% mothers in the SGA group versus 19.6% in the AGA group. The average length of prenatal stay was 2.3 (SD = 5.9, median = 0 and IQR = [0;2]) days in the SGA group versus 1.8 (SD = 5.3, median = 0 and IQR = [0;1]) days in the AGA group (*p* < 0.001). The average length of post-partum hospitalisation was higher in the SGA group, 5.9 (SD = 4.7, median = 5 and IQR = [4;6]) days versus 4.9 (SD = 3.4, median = 4 and IQR = [4;5]) days (*p* < 0.001).

The average pregnancy hospital costs were €868 (SD = €1,975, median = €0 and IQR = [€0; €932]) and €655 (SD = €1,736, median = €0 and IQR = [€0; €746]) in the SGA and AGA groups respectively for all mothers. The average delivery costs were €2,563 (SD = €1,443, median = €2,418 and IQR = [€2,033; €2,806]) and €2,357 (SD = €917, median = €2,076 and IQR = [€2,033; €2,418]) in the SGA and AGA groups respectively. The total average pre- and post-natal cost for mothers was higher in the SGA group €3,431 (SD = €2,637; median = €2,689 and IQR = [€2,123–€3,738]) versus €3,012 (SD = €2,138; median = €2,418; IQR = [€2,033–€3,127]) in the AGA group (*p* < 0.001). After stratifying by gestational age, the average total cost was higher in the SGA than in the AGA infants in all age groups (Table 4).

**Table 4:** Pregnancy to one year hospitalisation costs: mean (standard deviation), by gestational age for SGA and AGA infants

	Gestational age									
	[22–28[		[28–32[		[32–37[		[37–39[		[39–43[	
	*N* = 1,213		*N* = 3,346		*N* = 29,165		*N* = 101,974		*N* = 344,750	
	SGA	AGA	SGA	AGA	SGA	AGA	SGA	AGA	SGA	AGA
	*N* = 517	*N* = 696	*N* = 1,791	*N* = 1,555	*N* = 8,789	*N* = 20,376	*N* = 17,491	*N* = 84,483	*N* = 56,100	*N* = 288,650
	Mean (SD)	Mean (SD)	Mean (SD)	Mean (SD)	Mean (SD)	Mean (SD)	Mean (SD)	Mean (SD)	Mean (SD)	Mean (SD)
PREGNANCY (maternal)	€1,785 (€2,673)	€1,763 (€2,387)	€2,739 (€2,814)	€4,150 (€6,295)	€3,238 (€3,358)	€2,759 (€3,507)	€1,726 (€2,486)	€1,160 (€1,945)	€640 (€1,404)	€477 (€1,119)
DELIVERY (maternal)	€6,675 (€12,363)	€3,735 (€2,220)	€6,175 (€1,242)	€6,306 (€3,557)	€6,296 (€2,335)	€4,878 (€2,203)	€3,395 (€818)	€3,026 (€726)	€2,585 (€773)	€2,481 (€508)
INITIAL HOSPITALISATION (infant)	€42,539 (€33,838)	€36,905 (€30,188)	€28,671 (€14,143)	€21,935 (€14,680)	€8,484 (€7,869)	€4,539 (€5,313)	€2,067 (€2,620)	€1,074 (€1,315)	€1,170 (€1,374)	€979 (€849)
FIRST YEAR OF LIFE	€17,273 (€31,936)	€21,145 (€35,397)	€14,143 (€22,627)	€13,571 (€19,624)	€3,868 (€8,899)	€1,994 (€6,230)	€715 (€3,060)	€392 (€1,935)	€416 (€2,615)	€287 (€1,833)
TOTAL AVERAGE COST	€68,272 (€43,776)	€63,547 (€43,951)	€49,304 (€27,118)	€43,490 (€21,423)	€18,783 (€12,078)	€12,026 (€8,585)	€6,634 (€4,313)	€4,682 (€2,556)	€4,487 (€3,080)	€3,926 (€2,081)
	57,772	51,948	42,545	38,885	12,551	9,721	5,554	4,121	3,703	3,467
Median (IQR)	[38,780; 86,606]	[34,779; 81,310]	[30,175; 60,134]	[27726; 52,644]	[6,822; 22,688]	[6,330; 15,051]	[3,742; 8,324]	[2,909; 5,824]	[2,443; 5,628]	[2,476; 4,855]
Average cost difference between SGA and AGA infants	€ 4,725		€ 5,814		€ 6,757		€ 1,952		€ 561	

#### Infants

The 84,688 SGA births had 1.3 hospital admissions/infant during the first year, with a readmission rate of 16.4%, while the 395,760 AGA births had 1.2 admission/infant and a 12.9% readmission rate. There were also more emergency hospital transfers in the SGA group than in the AGA group (8 vs. 3.6%) and a higher proportion of infants transferred to intensive care during the initial or subsequent admissions (22.5% versus 8.3%, *p* < 0.001). The excess transfer and readmission rate was greater for SGA infants with preterm birth (31.3% versus 25.3% in preterm AGA, *p* < 0.001) than for full term births (14.2% versus 12.2% for term AGA, *p* < 0.001).

The average length of stay for the initial hospitalisation was 7 days (median = 4 and IQR = [4;6]) in the SGA group versus 4.6 days (median = 4 and IQR = [3;5]) in the AGA group (*p* < 0.001) with an average length of stay in intensive care of 3.4 days (median = 0 and IQR = [0;0]) versus 0.7 day (median = 0 and IQR = [0;0]) (*p* < 0.001). The average total length of stay during the first year was 7.8 days (median = 3 and IQR = [2;7]) versus 5.1 days (median = 3 and IQR = [2;6]), (*p* < 0.001).

The average cost of the initial hospitalisation was €2,948 (SD = €7,292; median = €903; IQR = [€903; €1,699]) in the SGA group and €1,328 (SD = €3,053; median = €903; IQR = [823; 903]) in the AGA group. The average costs by infant of all readmissions were €1,229 (SD = €6,167, median = €0 IQR = [€0; €0]) and €486 (SD = €3,245, median = €0 IQR = [€0; €0]) respectively. The total average cost for infants was €4,178 (SD = €10,711 median €1,205 IQR = [€903; €2,486]) in the SGA group and €1,814 (SD = €4,996; median = €903; IQR = [€903; €1,506]) in the AGA group. All cost differences were significant (*p* < 0.001) (Table 4). Medians and IQR at 0 are explained by situations were more than 75% was not hospitalized, and thus had a cost at 0.

#### Total cost

The total average cost for infants and mothers from the beginning of pregnancy through the first year of life was €2,783 higher in the SGA group than in the AGA group: €7,609 (SD = €14,041, median = €4,213 IQR = [€2,252; €7,666]) and €4,826 (SD = €6,255, median = €3,070 IQR = [€1,817; €5,422]) in the SGA and AGA groups respectively.

**Fig. 3:**
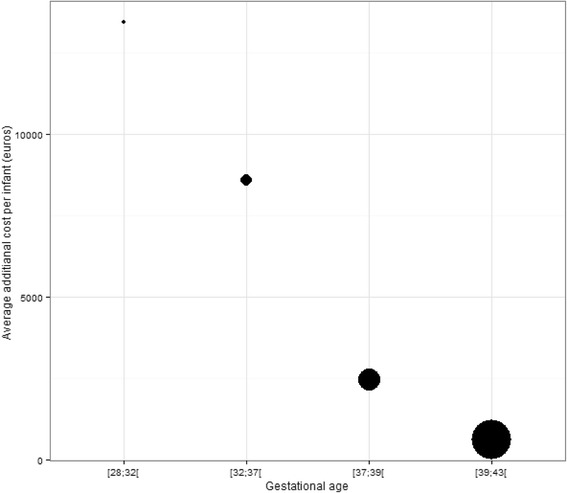
Hospital costs difference between SGA and AGA births according to gestational age

After stratifying newborns by gestational age, the average total cost for infants was higher in the SGA group than in the AGA group for all age groups. The highest cost difference was found in the [32-37 [gestational age group (*N* = 29,165 6%) (Fig. 3). The cost difference decreased with increasing gestational age (Fig. 3 and Table 4).

The total cost of pre-and post-partum (1 year) hospital admissions for SGA in France were €638 million. The additional cost for SGA compared to AGA was €1,478 million of which one-third (€493 million) was spent on preterm and two-thirds (€985 million) on full term SGA infants.

The total cost from pregnancy through the first year of life of all deliveries in France was €2,756 billion for 830,000 yearly births: SGA newborns represented 10.9% of total deliveries and 23% of total costs.

## Discussion

### Main findings

While there is published evidence on the economic consequences of preterm birth in several countries, the impact of being small for gestational age has seldom been assessed. Our main findings are that, of the total 779,376 births in France, the 84,688 SGA births cost on average €7,609 per infant (median = €4,213) from pregnancy to 1 year of age, or €2,783 more in average than AGA infants; SGA infants represented 23% of total delivery costs.

Regardless of the term, compared to AGA infants, SGA infants had longer hospital stays at delivery, were more likely to be admitted to an intensive care unit and were more likely to be hospitalised during the first year of life, resulting in higher costs. The mothers of SGA infants were also more likely to be hospitalised during pregnancy and had longer delivery stays. Total costs were highest for infants born between 22 to 28 weeks of gestation, and excess costs were highest for infants born between 32 to 37 weeks of gestation. After 37 weeks of gestation, the cost difference decreased as the gestational age increased. By contrast, the excess mortality increased when gestational age increased and was higher in late preterm and term infants than in infants at 28–36 weeks gestation.

Several reasons may explain this finding: the outcome difference for pre-term births may be driven mostly by gestational age rather than foetal growth, thus reducing the relative effect of SGA. Additionally, the absence of early diagnosis may have led to suboptimal obstetrical and neonatal management, which in turn may have resulted in low costs but poor outcome.

### Strength and limitations

The strength of our study lies in the fact that we have an exhaustive French hospital database with record linkage over a 2-year period. Non-hospital births and births with coding errors or imperfect linkage were excluded; the initial dataset comprised 779,376 births of the 792,996 live births registered. Hospital costs were calculated according to national French health insurance references. We also included maternal costs, which are seldom reported in perinatal economic studies.

Although the discharge database was initially developed as a tool for assessing funding related to medical activities in public and private hospitals, the main variables used in this study were recently found to be robust when compared with national vital statistics data [13] and to a national perinatal survey [10].

Furthermore, reporting of the variables in our study (length of stay, intensive care utilisation, birth weight, gestational age, mortality) are legally binding on hospitals and subject to controls. We chose ‘hyper normal’ infants for the comparator group but estimated the average neonatal cost for the population with the full dataset.

The main limitation was that our study considered only hospital costs. Moreover, the 1-year time horizon is too short to fully capture the economic consequences of SGA birth. The long term health impacts have been documented, and their costs will be assessed in future cohort studies.

### Interpretation

Other studies of preterm infants compared to full term infants found both higher mortality and complication rates [17], higher service utilisation and costs [4, 8] and an inverse relationship between gestational age and costs [6–8, 16]. The total costs of preterm infants in our study are comparable to those reported in countries with developed neonatal intensive care units [3, 4, 8, 9]. Full resuscitation is not standard in France before 24 weeks of gestation; palliative care is provided to infants born earlier. Thus costs in the 22–28 weeks gestational age group may be even higher in countries were full resuscitation is standard care. Lim found that on average the cost of SGA infants was twice the cost of AGA infants [2]. This cost difference is higher than in our study but used a different costing methodology. In contrast to the Canadian study [2], we found that the difference between AGA and SGA hospital costs was strongly related to gestational age, decreasing monotonously from 28 to 32 weeks gestational age to term infants. The rates of caesarean sections in both preterm and SGA in Canada were markedly lower (6–13%) than in France [2, 18]. The most recently published cost study of preterm infants in the East Midlands reports birth to discharge hospital costs comparable to ours, with the same association between costs and gestational age [6].

## Conclusion

We found that being SGA predicted high hospital costs regardless of gestational age. The additional cost of SGA births was explained by higher complication rates, more frequent hospital readmissions and longer lengths of stay. SGA appears to be an independent contributor to high hospital costs in the short term. This data could be used in models to predict the costs associated with SGA [19].

## Abbreviations

AGA: Appropriate for gestational age
CNIL: Commission nationale de l’informatique et des libertés
DRG: Diagnostic Related Group
ICD-10: International Classification of Diseases 10th Revision
IQR: Inter-Quartile Range
PMSI: Programme de Médicalisation des Systèmes d’Information
SD: Standard Deviation
SGA: Small for Gestational age
